# Entropy based analysis of SARS-CoV-2 spread in India using informative subtype markers

**DOI:** 10.1038/s41598-021-95247-5

**Published:** 2021-08-05

**Authors:** Piyush Mathur, Pratik Goyal, Garima Verma, Pankaj Yadav

**Affiliations:** 1 Department of Bioscience & Bioengineering, Indian Institute of Technology, Jodhpur, 342037 Rajasthan India; 2 Department of Experimental Medicine, System Biology Group, University La Sapienza Università di Roma, Roma, Italy

**Keywords:** Computational biology and bioinformatics, Genetics, Diseases

## Abstract

India became one of the most COVID-19 affected countries with more than 4 million infected cases and 71,000 deaths by September 2020. We studied the temporal dynamics and geographic distribution of SARS-CoV-2 subtypes in India. Moreover, we analysed the RGD motif and D614G mutation in the spike protein of SARS-CoV-2. We used a previously proposed viral subtyping method based upon informative subtype markers (ISMs). The ISMs were identified on the basis of information entropy using 94,515 genome sequences of SARS-CoV-2 available publicly at the Global Initiative on Sharing All Influenza Data (GISAID). We identified 11 distinct positions in the SARS-CoV-2 genomes for defining ISMs resulting in 798 unique ISMs. The most abundant ISM in India was transferred from European countries. In contrast, the second most abundant ISM in India was found to be transferred via Australia. Moreover, the eastern regions in India were infected by the ISM most abundant in China due to geographical linkage. Our analysis confirmed higher rates of new cases in the countries abundant with S-G614 strain compared to countries with abundant S-D614 strain. In India, overall S-G614 was most prevalent compared to S-D614, except a few regions including New Delhi, Bihar, and Rajasthan.

## Introduction

The first case of large-scale infectious pneumonia was reported in Wuhan, China, in December 2019. Later, within a few months, several other countries started notifying the confirmed cases of similar infection named by world health organization (WHO) as COVID-19^1^. The virus responsible for COVID-19 was named SARS-CoV-2 due to its genetically relatedness to severe acute respiratory syndrome coronavirus (SARS-CoV). By the time of this study, around 26.7 million diagnosed cases have been reported worldwide and more than one million deaths have occurred^2^.

The novel coronavirus has undergone many rapid mutations as it started spreading around the globe. These mutations can affect the way viruses infect cells and replicate within them. They can lead to subtle changes in viral proteins, which can be very helpful in preventing the existing antibodies in the immune system from recognising the virus. Mutations can also reduce the efficiency of antiviral treatments. Recently, it was reported that 22 subtypes of SARS-CoV-2 has a much lower mutation rate in comparison to other SARS viruses^3^. Though subtle mutations in SARS-CoV-2 are of less concern, yet they help in tracking the spread of virus across populations. The mutational patterns can predict an expected second wave of COVID-19 pandemic. Furthermore, understanding the mutational chronologies might assist in vaccine development.

The entry of SARS-CoV-2 into host is mediated by spike glycoprotein (or S-protein) which is considered as the main target for development of vaccines and therapeutics. It has been analysed that the gene coding for S-protein does not mutate very frequently and are observed to lie in conserved region. However, one dominant mutation at position 614 of the S-protein (i.e., D614G mutation) is known to affect viral infectivity. S-G614 pseudovirus infects 293 T-ACE2 cells significantly more efficiently than the S-D614 pseudovirus, especially in the presence of elastase-2 ^4^. A study showed that the S-protein of SARS-CoV-2 produced an evolutionary mutation of K403R compared with the S-protein of SARS-CoV, thus forming an adjacent RGD motif at the interaction surface^5^. The RGD motif is considered as a ligand for many cell surface integrins. Several studies reveal that the binding of S-protein of SARS-CoV-2 with integrins facilitate the infection process of the virus^5^.

In present study, we deploy a genetic subtyping method in order to identify the potent informative subtype markers (ISMs) in genome and use them for subtyping^6^. This method exploits information entropy as a measure to identify highly informative genetic variation sites termed as ISM and differentiate between the SARS-Cov-2 subtypes^6^. This study is focused on the progression of COVID-19 in India. In particular, we study the geographical distribution of different viral subtypes across India and identify the countries with viral subtypes similar to those found in India. Further, we investigate how specific viral subtypes have spread across the world in a time dependant manner. Moreover, we perform geographical distribution and temporal dynamics analysis for the D614G mutation in order to understand the spread before and after mutation. Further, we investigate the progression of the major two strains (S-614G and S-614D) in different regions in India and other countries. Moreover, we investigated the locus coding for conserved RGD motif in the S-protein.

## Materials and methods

### Datasets

We used whole gnome nucleotide sequences data of SARS-CoV-2 from 94,541 samples across the world collated at the Global Initiative on Sharing All Influenza Data (GISAID; URL: https://www.gisaid.org/) as on September 7th, 2020^7^. The reference nucleotide sequence with accession number NC_045512.2 was retrieved from National Center for Biotechnology Information (NCBI) for the purpose of sequence alignment^8^. In order to track the total number of COVID-19 cases with time, the data was collected from the *Our World in Data*^9^.

The ISM framework used for performing genetic subtyping is previously available at the GitHub repository (https://github.com/EESI/ISM).

### Data pre-processing

Firstly, the nucleotide sequences of length either < 25 or > 31 kilobases (kbs) were removed from further analysis. The remaining 94,515 sequences were aligned by using the *MAFFT* tool v7.471^10^. This multiple sequence alignment tool uses a rapid calculation of full-length multiple sequence alignment of closely related viral genomes with a reference sequence by adding “auto” option and threading using “thread-1”. This resulted in multiple aligned sequences of length 54,053 bases. The multiple aligned sequences were combined with accompanying metadata available at the GISAID. The metadata includes country of origin of patient sample, submitting laboratory, sample collection and submission date. We removed sequences of non-human hosts and sequences with incomplete collection dates. The remaining 91,568 sequences were considered for the entropy-based analysis.

### Entropy analysis

The entropy is a measure of uncertainty and can be used to find out positions (or sites) with high mutations. We calculated the information entropy of all the positions using the aligned sequences. The sites with high entropy were treated as ISM. A few ambiguous bases (or noises) were present in the sequences namely, *B*, *D*, *H*, *K*, *M*, *Y*, *R*, *S*, *V*, *W* and *N*. The gaps introduced in the sequences after performing MSA were notated with a dash (–) symbol. The information entropy of any *n*th position in the genome can be computed using equation:

1 $${H}_{n}\left(X\right)=\sum {P}_{n}\left({x}_{i}\right){\mathrm{log}}_{2}{P}_{n}({x}_{i})$$

where, *X* is a random variable with possible values *x*_*i*_ referring to all bases including the ten ambiguous bases at the *n*th position. The ${P}_{n}\left({x}_{i}\right)$ refers to the frequency of occurrence of a base *x*_*i*_ at the *n*th position.

For the purpose of this study, we used a modified measure of information entropy known as masked entropy which can be computed with the above equation. Here, *x*_*i*_ refers to all the bases including the ambiguous bases at the *n*th position except base N and dash (–). This is due to the fact that sites with high number of *N* or dash (–) provide least information (or high mutation) and cannot be included in ISM formation. In order to form ISM, the distribution of masked entropy and the null frequency was calculated across the sites. Here, the null frequency refers to frequency of base N or dash (–) at a position in the multiple aligned sequences divided by the total number of sequences. The sites with an entropy ≥ 0.4 and null frequency < 0.25 were selected to form ISMs. Thus, a specific number of distinct sites were selected and traced in all the sequences to form an ISM. Similarly, the ISM for the reference genome sequence was formed (i.e., reference ISM).

### Informative subtype markers (ISMs) grouping

We corrected for the ambiguous symbols (noise) in the ISMs. To this end, ISMs were corrected either completely or partially depending upon the amount of ambiguity. Next, we performed hierarchical clustering on the 50 most abundant ISMs using the *hierarchical clustering* package in SciPy library^11^. We used the linkage function in this package with “average” method which applies the UPGMA algorithm for linkage^12^. For this purpose, pairwise hamming distances was calculated for the 50 most abundant ISMs. The hierarchical clusters were visualised using the dendrogram function of the same package along with *matplotlib.pyplot* package.

Most abundant ISMs were used for geographical distribution and temporal analysis of SARS-CoV-2 subtypes. In order to study the geographical distribution of viral subtypes, we used pie charts to show the relative abundance of different viral subtypes in each region. Each unique ISM refers to a specific SARS-CoV-2 subtype. For purpose of convenience, we used a global color map to assign unique color to each viral subtypes. Throughout the study, we used same viral subtype specific color in order to make it easier to analyse the temporal and geographical analysis of an ISM. We performed geographical analysis in different countries and particularly focussed on regions specific analysis of India.

### Temporal analysis

The temporal analysis shows the progression of the SARS-CoV-2 subtypes as a function of time. The dates associated with the sequences is used to find out the relative abundance of the viral subtypes in a country through the course of time since a given point of time. The relative abundance *ISM*_*(s,c)*_*(t)* of a subtype *s*, in a region *c* at a given point of time *t,* is calculated as:

2 $${ISM}_{(s, c)}(t) = \frac{{ N}_{(s,c)}(t)}{{N}_{c}(t)}$$

where, *N*_*(s,c)*_*(t)* indicates total number of instances of subtype *s* in a region *c* till a point of time *t*, and *N*_*c*_*(t)* is the total number of sequences in the location *c* till a point of time *t.*

### Phylodynamic and phylogeographic analyses

We used the 798 ISMs to perform phylodynamic and phylogeographic analyses using BEAST2 tool^13^. The dates of first appearance were used as tip dates for these ISMs. The latitudes and longitudes of the countries were used as location points for phylogeographic analysis^14^. Supplementary document describes the details of how these analyses were performed. The final phylodynamic and the phylogeographic plots were generated using FigTree v1.4.4 and SPREAD v1.0.6 respectively^15,16^.

### D614G mutation

We identified the position of the D614G mutation in the multiple aligned sequences using the reference genome sequence. Next, we mapped the coded amino acid at 614 position. The dash (–) symbol indicates a gap at the 614 position in a sequence. The geographical distribution of the two major strains (S-614G and S-614D) was shown using pie charts. As mentioned earlier, throughout the study, we used a constant global color map for these two strains. The temporal analysis of relative abundance of the two strains across different countries was performed as described above.

## Results

### ISMs formation

**Figure 1:**
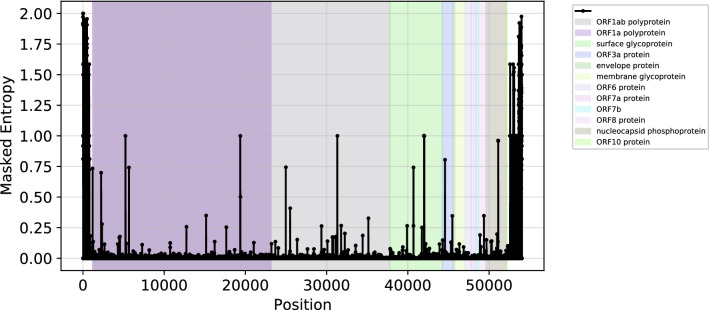
Shows the masked entropies at different genomics positions in the multiple aligned genome sequences of SARS-CoV-2. The different colors indicate coding regions of the genome. The untranslated regions (UTRs) are shown in white color. Note that the two polyproteins ORF1ab and ORF1a are overlapping because both are coded by the same ORF1ab gene.

The masked entropy of all the positions in the multiple aligned genome sequences of SARS-CoV-2 was calculated using the Eq. (1). Figure 1 shows the masked entropy of all the positions. Two proteins namely, ORF1ab and ORF1a are overlapping because both these are coded by ORF1ab gene. The ORF1a region lies between 1.2 kb and 23.1 kb, while ORF1ab lies between 1.2 kb and 37.8 kb. The three codons coding for the RGD motif were found to have negligible masked entropy (see Supplementary Table 1). The masked entropies of different positions in the spike glycoprotein region of the SARS-CoV-2 genome is shown in Supplementary Fig. 1. We found only a few positions in the spike glycoprotein region with high masked entropy (entropy > 0.4). Among these, the position 40,689 with masked entropy equals to 0.742 was selected for constructing ISMs. Note that positions near 42,000 also showed high entropy, but they are not selected for ISM formation owing to their high null frequency. The histogram distribution of masked entropies and null frequencies across different positions are shown in Supplementary Fig. 2. The null frequency threshold equals to 0.25 was chosen on the basis of null frequency distribution (see Supplementary Fig. 2b). Further, we chose masked entropy threshold of 0.4 to select positions for the formation of ISMs. Thus, 11 different highly informative positions were selected for the formation of ISM. Table 1 shows these 11 positions along with their masked entropy values and proteins coded at each respective loci. Interestingly, there is 1 position from the 5’ UTR and 5 positions from ORF1ab gene. Moreover, there are 3 positions from Nucleocapsid phosphoprotein coding region, 1 position each from ORF3a and Surface glycoprotein coding regions. In order to constitute the ISMs for each genome, we traced these 11 positions in the 91,568 genome sequences available from GISAID. Overall, 798 unique ISMs were identified.

**Table 1:** Shows 11 positions in the SARS-Cov2 genome selected for constituting informative subtype markers (ISMs).

ISM index	MSA position	Reference position	Masked entropy	Protein(s)
1	1181	242	0.733	5′ untranslated region
2	2235	1060	0.698	ORF1ab polyprotein, ORF1a polyprotein
3	5665	3038	0.741	ORF1ab polyprotein, ORF1a polyprotein
4	19,376	11,084	0.502	ORF1ab polyprotein, ORF1a polyprotein
5	24,968	14,409	0.744	ORF1ab polyprotein
6	25,499	14,806	0.409	ORF1ab polyprotein
7	40,689	23,404	0.742	Surface glycoprotein
8	44,576	25,564	0.805	ORF3a protein
9	51,122	28,882	0.962	Nucleocapsid phosphoprotein
10	51,123	28,883	0.960	Nucleocapsid phosphoprotein
11	51,124	28,884	0.958	Nucleocapsid phosphoprotein

*ISM index* ISM position index, *MSA position* base-pair position in the reference genome post multiple sequence alignment, *Reference position* base-pair position in the reference genome sequence, *Masked entropy* masked entropy value at respective ISM position, *Protein(s)* protein(s) encoded by the gene in this position.

### Hierarchical clustering of ISMs

**Figure 2:**
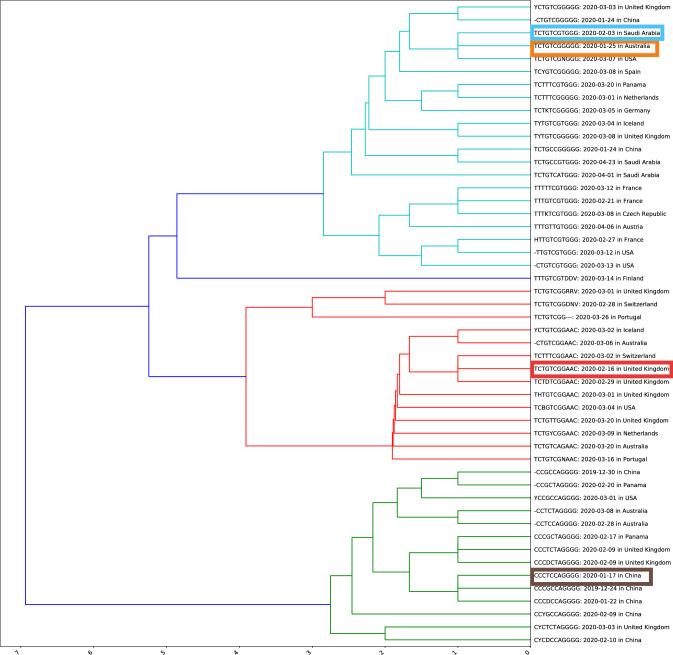
Hierarchical clustering of top 50 most abundant unique ISMs. The four most abundant viral subtypes in India are highlighted as thick rectangles in red (*TCTGTCGGAAC*; 25.7%), yellow (*TCTGTCGGGGG*; 20.7%), blue (*TCTGTCGTGGG*; 18.3%) and brown (*CCCTCCAGGGG*; 14.7%). The dates of first sampling along with the country name is shown for each ISM. Note that the ISM *CCCTCCAGGGG *(in brown rectangle) was sequenced in China on January 17th, 2020.

We performed hierarchical clustering of 50 most abundant unique ISMs based on pairwise hamming distances (see Fig. 2). In the dendrogram, each ISM is shown with their date and country of the earliest instance. The four top most abundant ISMs found in India are highlighted with different colors namely, red (*TCTGTCGGAAC*; 25.7%), yellow (*TCTGTCGGGGG*; 20.7%), blue (*TCTGTCGTGGG*; 18.3%) and brown (*CCCTCCAGGGG*; 14.7%). We compared the relative abundance of ISM *TCTGTCGGAAC* in different countries (Supplementary Fig. 4). We found that this subtype was also highly abundant in several countries including Australia, Russia, Japan, Brazil, Italy, Bangladesh, and South Africa. Interestingly, the two most abundant ISMs of China, namely *CCCGCCAGGGG* (blue color) and *CCCTCCAGGGG* (yellow color) are found to be similar in sequence and close to fourth most abundant ISM of India viz *CCCTCCAGGGG* in brown color (see Fig. 2, green cluster). Thus, indicating that this ISM might have been a direct transfer from China to India. Supplementary Fig. 3 shows an exponential increase in the number of Covid-19 cases as the mutations rate became higher. The hamming distance was computed for the 50 most abundant ISMs with the reference ISM. The number of cases shows a direct relationship with the hamming distances between ISMs.

### Geographical distribution of ISMs

**Figure 3:**
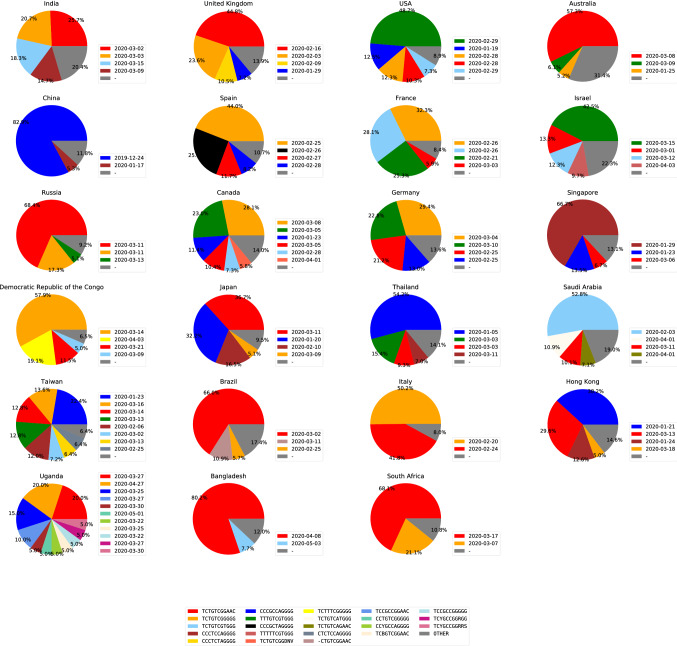
Shows the geographical distribution of viral subtypes across different countries. The boxes beside the pie chart indicates the first sampling date of ISMs in that country. The legend at the bottom shows the color map for each ISM.

For our convenience, we used a collective global color map to assign specific color code for each ISM (see Supplementary Fig. 5). This color map of the ISMs is used in the geographical and temporal analysis. The geographical distribution of various viral subtypes across different countries is shown in Fig. 3. Similarly, the geographical distribution of these viral subtypes in different regions of India is shown in Supplementary Fig. 6.

### Temporal analysis of ISMs

**Figure 4:**
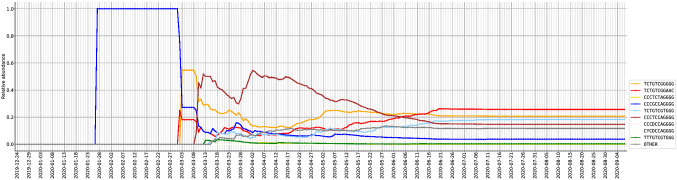
Temporal analysis show the relative abundance of different viral subtypes in India over a period of time. The lines become horizontal after a point of time because sequences sampled after that time haven’t been submitted as of the retrieval date. The two light colored ISMs *CCCDCCAGGGG* and *CYCDCCAGGGG* were found to be abundant in Supplementary Fig. 6.

To study the progression of the SARS-CoV-2 subtypes in the time domain, we estimated the relative abundance of viral subtypes in the country over a time period. Figure 4 shows the relative abundance of different viral subtypes found in India as sampled over time. Notably, the subtype corresponding to top ISM (*CCCGCCAGGGG*; blue color) from China was the first to infect Indian population. Though this subtype is not highly abundant in India, because it might have incurred several mutations. This ISM has a local peak around March 28th, 2020 and last peak around May 7th, 2020 referring to sampling reported in Madhya Pradesh and Odisha, respectively. After this, it follows a decline in its abundance indicating no more cases of that viral subtype. The red ISM has a very uniform increase in abundance throughout unlike other ISMs which show sudden peaks and drops in abundance. This can be because this ISM was abundant in most of the regions in India (see Supplementary Fig. 6). The two light colored ISMs *CCCDCCAGGGG* and *CYCDCCAGGGG* which were found to be abundant in Supplementary Fig. 6 can be seen at the bottom in Fig. 4. This indicate that unlike blue and brown ISMs, these two ISMs did not reach a high peak even for a small time period. This suggests that these two viral subtypes were only abundant in New Delhi. Note that there are no changes after end of June, because our study included samples submitted by September 7th, 2020.

### Phylodynamic and phylogeographic analysis

As a result of the phylodynamic analysis, we plotted the maximum clade credibility (MCC) tree using FigTree with the calculated time of the respective nodes which is shown in Supplementary Fig. 7. The figure shows calculated time to all the common ancestor nodes. It shows the time to the root of the tree as 2019.4383 (June 9th, 2019) which is referred to as the time to the most recent common ancestor (TMRCA) with HPD 95% (95% highest posterior density interval). In addition to this, we also performed phylogeographic analysis which is shown in Supplementary Fig. 8. The figure shows the branches of the MCC tree for phylogeographic analysis which have their color from black to red according to their node height. The circular polygons shown in cyan color refers to a discrete state in the MCC tree where the radius and color (black to cyan) indicate the number of lineages holding that discrete state at a time. The regions majorly highlighted by circular polygons are marked by their country names.

### D614G mutation analysis

**Figure 5:**
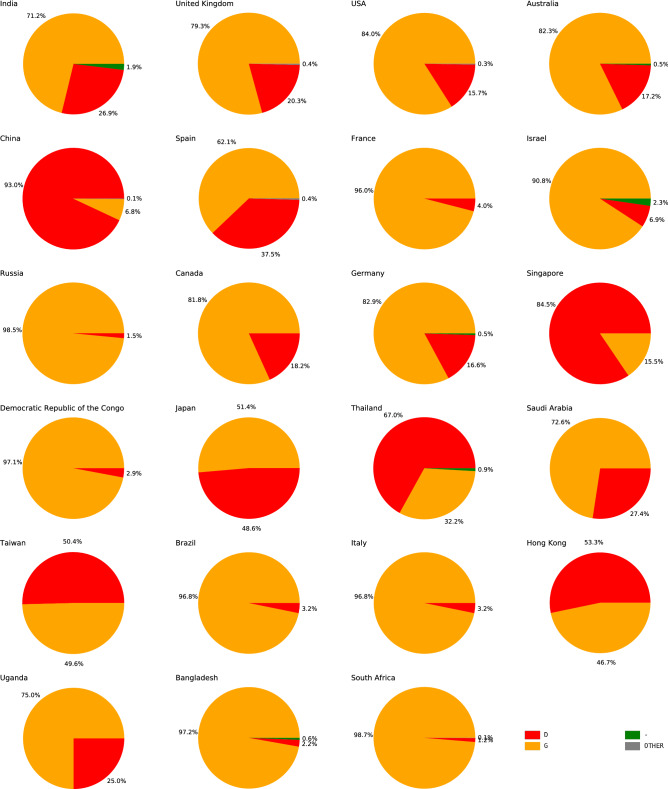
Shows the geographical distribution of two strains S-D614 and S-G614 across different countries. The red and yellow colors refers to S-D614 and S-G614 strains, respectively. The ‘OTHER’ refers collectively to the strains with relative abundance less than 0.5%. The green color and dash (−) symbol refers to the non-coding amino acid at the position 614 of S-protein.

The geographical distribution of the two strains S-D614 and S-G614 across different countries is shown in Fig. 5. In majority of the countries G-strain is highly abundant except for eastern and south-eastern Asian countries. This indicates SARS-CoV-2 underwent an important D614G mutation to infect the population in other regions with different climatic conditions and diet pattern. Interestingly, Spain is the only non-Asian country with a fairly high abundance of D-strain. Further, the geographical distribution of different strains across different regions in India is shown in Supplementary Fig. 9. The progression of S-D614 and S-G614 as well other strains in the time domain was analysed by plotting the relative abundance of the strains in the country against time (Supplementary Fig. 10).

Moreover, we compared G-strain abundant countries with D-strain abundant countries using data from the Worldometer (https://www.worldometers.info/coronavirus/)^17^. The plots of daily Covid-19 cases with time for all the D-strain abundant countries except China, owing to the fact that it was the first country affected by the virus, already had a peak in March or April month, which was followed by a decline in the daily cases (data accessed on October 9th, 2020). Among the D-strain abundant countries, most had a second peak in the second week of August 2020, whereas for a few G-strain abundant countries second peak was at beginning of month May, 2020. One major difference in these two groups of countries was that the D-strain abundant countries had lesser number of daily cases compared to G-strain countries. In India, the daily new cases showed a rise at beginning of May, 2020 which is around the same time when G-strain became more abundant than D-strain (Supplementary Fig. 10). Moreover, in India, the daily cases showed a peak around mid-September, 2020.

## Discussion

We studied the various SARS-CoV-2 subtypes and their geographical distribution particularly in India. To this end, we identified the most abundant viral subtype occurring in India. Further, we identified countries which might be linked to the Indian isolates of the virus. We studied how regional clusters are related to each other and which viral subtype is dominant in a given region and trace their source of origin.

The masked entropy of all the sites in the aligned genome sequences were computed to form ISMs (see Fig. 1). We found several sites in the 5’ UTR and 3’ UTR in the genome which show high entropies (i.e., entropy > 1.0). This is because the MSA introduces gaps at the ends of the genomes to align the sequences as genome sequencing is inaccurate at ends^18,19^. Interestingly, the ORF1ab gene (from 266 to 21,555 bases) codes for two polyproteins ORF1ab and ORF1a, but only a few of the sites in this gene have high entropy (i.e., entropy > 0.4). It also has a region from 5 to 12 kbs with very less masked entropy (i.e., entropy < 0.2; see Fig. 1). Another gene named *surface glycoprotein* in a conserved region represent only one site with high uncertainty. Furthermore, genes coding for envelope protein, membrane glycoprotein, ORF6 protein, ORF7a protein, “ORF7b” and “ORF10 protein” are also conserved. This helps in identifying constant regions of SARS-CoV-2 genome and regions of uncertainty for selection of sites for ISM. Among the 11 sites used for making ISMs, one site is from the 5’ UTR and five sites are from gene “ORF1ab” (see Table 1). There are three sites coding for “nucleocapsid phosphoprotein, one for “ORF3a protein” and one for “surface glycoprotein”. These highly mutating sites can be used for tracking the viral subtypes. Further, a few of actively mutating sites may be affecting functions of virus. The gene coding for nucleocapsid phosphoprotein is 1260 bp long and has three consecutive sites in ISM, informing the fact that this region is actively mutating as compared to other regions. Further, the ORF1ab gene has five sites in the ISM, but this gene is 22 kb long making it difficult to conclude about its mutating activity. However, it might be possible that a few regions in this gene are actively mutating. Notably, we found negligible masked entropy of the nine sites in the 3 codons region encoding for RGD motif (see Supplementary Table 1). This implies that the RGD motif is highly conserved in SARS-CoV-2. Previous studies reported that the S protein of SARS-CoV-2 produced an evolutionary mutation of K403R compared with the S protein of SARS-CoV, forming an adjacent RGD motif at the interaction surface^5^. This possibly indicates that RGD motif was one of the important mutations which enabled coronavirus from wild animals to infect humans. We also noticed an exponential increase in the number of Covid-19 cases as the number of ISMs increased (Supplementary Fig. 3). Moreover, as the number of ISMs and COVID-19 cases increase, the hamming distances of the ISMs with reference sequence also increase. The co-dependency of mutations and number or COVID-19 cases might be a possible explanation for such a behaviour. The frequent mutation causes SARS-CoV-2 to be more infectious which increases the number of COVID-19 cases. In turn, more infections allow SARS-CoV-2 to mutate more.

Our hierarchical clustering analysis identified top 4 ISMs in India namely, *TCTGTCGGAAC*, *TCTGTCGGGGG*, *TCTGTCGTGGG* and *CCCTCCAGGGG* with relative abundance of 25.7%, 20.7%, 18.3% and 14.7%, respectively (Fig. 2). Interestingly, we identified an ISM *TTTGTCGTDDV* unique to Finland (blue cluster). In the green cluster, the ISMs found were those sequenced in early stages of onset of Covid-19 in the China and also the ISMs which may have been transmitted later to other countries. Within the same cluster, we found an ISM abundant (14.7%) in India which was first sequenced in China on January 17th, 2020 (Fig. 2; brown bold lines). In addition, this cluster has three sub-clusters. First sub-cluster has ISMs sequenced in early stages in China which later reached the United Kingdom. Second sub-cluster has ISMs sequenced in later stages in other countries. Third sub-cluster has ISM sequenced later in China. In the red cluster, the ISMs were sequenced first mostly in European countries in February and March 2020. A few ISMs in this cluster were also sequenced first in the USA and Australia. This clearly indicated that the viral subtypes corresponding to these ISMs were transferred to those countries from Europe. Since the first sequenced ISM in this cluster was sequenced in February 2020, indicating that after entering the European countries SARS-CoV-2 incurred many mutations. This explains at least in part why patients in European countries were heavily affected with severe symptoms. Within same cluster, one ISM (bold red lines) abundant in India marked red which was first sequenced in the United Kingdom on February 6th, 2020. In the cyan cluster, the ISMs were sequenced first in the later stages of the pandemic, i.e., March and April 2020, mainly in European, American and Asian countries. In this cluster, one ISM was first sequenced in Australia on January 25th, 2020. Majority of ISMs are very close in this cluster, indicating that the viral subtypes corresponding to ISMs of this clusters have been transmitted to other countries from Australia. Two ISMs (yellow and blue bold lines) abundant in India marked were sequenced first in Australia on January 25th, 2020 and in Saudi Arabia on February 3rd, 2020. This indicates that the viral subtype corresponding to ISM *TCTGTCGGGGG* might be transferred to Saudi Arabia from Australia. Later, this subtype incurred mutations resulting in viral subtype corresponding to ISM *TCTGTCGTGGG* which spread to Asian populations.

The top most ISM of India, *TCTGTCGGAAC*, was highly abundant in several other countries including Australia, Russia, Japan, Brazil, Italy, Bangladesh, and South Africa (Supplementary Fig. 4). Notably, this subtype was firstly sequenced in the United Kingdom (Fig. 2; red cluster). This suggest that other countries received this subtype from European countries. Conversely, Bangladesh received this viral subtype from India as it was sequenced earlier in India (Fig. 3). Furthermore, date of sequencing suggests that in other countries this subtype may have been transmitted from either United Kingdom or some other European country.

Recent reports claimed that migration of Indian students from Singapore on March 19th, 2020 led to transmission of the Covid-19 in India^20^. However, our study shows that the speculated viral subtypes corresponding to red and brown ISMs were sampled in India even before the arrival of students (see Figs. 3 and 4). Rather, our study show that migration of some 400 stranded Indians from Bangladesh and Singapore on March 6th, 2020 could have affected the Covid-19 outbreak in India^21^. Because the viral subtype shown in brown color (*CCCTCCAGGGG*) was sampled in India on March 9th, 2020 and on January 29th, 2020 in Singapore with relative abundance of 66.7% in Singapore.

The two similar viral subtypes corresponding to *TCTGTCGGGGG* (yellow color) and *TCTGTCGTGGG* (light blue color) occurred abundantly in India (Fig. 3). From the dates of these ISMs shown in Figs. 3 and 2 (cyan cluster), it can be inferred that the former subtype (yellow) might have transferred to Asian countries and mutated to latter (light blue). Similarly, India could have received the yellow subtype from other Asian countries while light blue subtype from European countries. Overall, the dominant subtypes in India might have been transferred from mostly European and Asian countries. Moreover, a few subtypes might have been transferred from India to other countries such as Bangladesh.

Our region-based analysis in India show that the most abundant viral subtypes are common to most states (Supplementary Fig. 6). The top ISM of China was found to be highly abundant in Odisha, West Bengal and Madhya Pradesh (*CCCGCCAGGGG*; blue color). Among these regions, this subtype was first sampled in Madhya Pradesh. Thus, Madhya Pradesh was infected by this subtype from China because this ISM has low abundance in many countries (see Fig. 3). Further, owing to lock-down across the nation, this subtype might have been transferred slowly to eastern states. Interestingly, ISMs with low abundance in most states except New Delhi, Tamil Nadu and Bihar has low abundance in most countries (Fig. 3). It can also be seen that New Delhi has a significant percentage of viral subtypes contributing to ‘OTHER’ subtypes which means that it has a fairly large number of different viral subtypes. Being the capital of India, the New Delhi has comparatively more travellers than other regions of the country. Thus, diverse viral subtypes from other regions of India might have transferred to Delhi, but not particular subtype could become dominant at least by the time of this study.

Our temporal analysis shows that the viral subtype corresponding to a top ISM from China *CCCGCCAGGGG* was the first subtype that infected Indian population (Fig. 4; blue color). However, this subtype has low abundance in India likely due to early imposition of country-wide lockdown. Moreover, we studied the role of D614G mutation in transmission of the infection. We can see that most of the countries in the figure show have G-strain as more abundant strain. This can be because D614G is a dominant mutation which increases the infectivity of the virus. The D-strain was more abundant in eastern and south-eastern Asian countries except Spain. In majority of the regions in India G-strain is abundant (Fig. 5). However, regions like Delhi, Rajasthan and Bihar show high abundance of D-strain (Supplementary Fig. 9). In March, 2020, Delhi and Rajasthan were among the regions which had many active cases of COVID-19 and D-strain was more infective during that time. The shifting towards G-strain points toward an adaptation in the virus to the changed weather conditions. Interestingly, comparison of countries with G-strain and D-strain showed that the D-strain abundant countries had lesser number of daily cases compared to G-strain countries. However, death rates of many of the D-strain abundant countries were similar to G-strain abundant countries. This may owe to facilities provided and measures taken by various countries are different. Thus, D614G mutation increases the infectivity, albeit the mortality rate remained stable across countries.

Our phylodynamic analysis of the 798 unique ISMs which is shown in Supplementary Fig. 7 states the TMRCA as June 9th 2019. This TMRCA value is less as compared to October 19th, 2019 as reported earlier^22^. Other studies on mapping of genetic variation of SARS-CoV-2 also show TMRCA between November to December 2019^23,24^. This difference in TMRCA may be due to mainly two reasons. Firstly, we have used ISM sequences instead of full genome sequences of SARS-CoV-2. Secondly, we chosen the date of first appearance for each ISM identified in our analysis, which might have possibly shifted the TMRCA value. Similarly, the phylogeographic analysis shown in Supplementary Fig. 8 indicates the origin of SARS-CoV-2 in United Kingdom instead of China. This could be possibly because of the first appearance of most ISMs (229 out of 798) in United Kingdom.

Our study relied on data available from GISAID which collected from only sparse regions of the world. The representation of sequences is not well distributed across locations and time. This limits our insights about how the different viral subtypes have spread in different regions and how they are linked to each other by looking at region-wise temporal analysis. For instance, Ladakh region showed 100% of ISM *CCCTCCAGGGG* (Supplementary Fig. 6; brown color). This can be explained by the unavailability of sufficient number of samples sequenced from this region. Further, longer ISMs were not considered because that will add irrelevant ISMs which are not very different from each other. This may bring down the efficiency of the subtyping method as well as significantly increase the computational cost owing to its high computational complexity. Further, ambiguous bases were corrected before use in our study. For instance, ambiguous base Y means the base can be either C or T. Thus, an ISM *ATTTATACCGY* will have same hamming distance with *ATTTATACCGC* as well as *ATTTATACCGA*. The proposed subtyping method has certain limitations. It only uses the location of the GISAID sequence provided in the metadata and not the location of exposure in subtyping. Moreover, the subtyping method relies on the masked entropy and null frequency thresholds and a change in these thresholds provides us with deeper or shallower subtypes. Hence, it might change the inferences about transmission of SARS-CoV-2 with different thresholds of masked entropy and null frequency. The dendrogram used in this study does not explicitly reveal the origin of ISMs. However, the entropy-based method used in our study is computationally efficient and can identify subtypes without relying on evolutionary model assumptions^6^. Conversely, the phylogenetic tree used to understand evolution of the virus on a macro and micro level are usually complex constructs and leads to addition of long branches due to minor errors which could create major difference in inferences^25^.

In summary, we identified viral subtypes clusters and tried to understand which regions might have been affected by the same or similar viral subtypes. We investigated how certain events may be responsible for the spread. Thus, our work provides insights in how extensively the virus might have mutated owing to climatic conditions or other factors. Most abundant SARS-CoV-2 strains in India were found to be indirectly transferred from China. Of these, the most abundant viral subtype in India was transferred from European countries. In contrast, the second most abundant subtype in India was found to be transferred via Australia. Moreover, the eastern regions in India were infected by the ISM most abundant in China due to geographical linkage. Our study identifies four major subtypes of SARS-CoV-2 in India which remained undetected in earlier phylogenetic studies. Our findings provide new insights about the transmission of SARS-CoV-2 in India and beyond. Thus, our work contributes to the field of genetic epidemiology and helps with countermeasures to limit the spread of SARS-CoV-2.

## Supplementary Information

Supplementary Information 1.

Supplementary Information 2.
