# Supplementary material for: Entropy based analysis of SARS-CoV-2 spread in India using informative subtype markers
Source: Sci Rep. 2021 Aug 5;11:15972. doi: 10.1038/s41598-021-95247-5 (PMC8342543; doi:10.1038/s41598-021-95247-5)
Supplement: Supplementary file 1 — Supplementary Information 1. [file 41598_2021_95247_MOESM1_ESM.docx]

**SUPPLEMENTARY DATA**

**Entropy based analysis of SARS-CoV-2 Spread in India using Informative Subtype Markers**

Piyush Mathur^1^, Pratik Goyal^1^, Garima Verma^2^, Pankaj Yadav^1^

1. Department of Bioscience & Bioengineering, Indian Institute of Technology, Jodhpur, Rajasthan, India
2. Department of Experimental Medicine, System Biology Group, University La Sapienza Università di Roma, Roma, Italy

**Materials and Methods**

*Spike protein entropy analysis*

The spike glycoprotein region has been closely observed. The masked entropy values used for **Figure 1** have been used to take a closer look at the spike glycoprotein. We have plotted the masked entropy values against their positions only for the glycoprotein region in **Supplementary Figure 1**.

*RGD motif Analysis*

We have observed the masked entropy values of the RGD motif in the spike glycoprotien. Firstly, the original reference genome sites are mapped with the sites in the reference genome after the MSA. Then the range for spike glycoprotein mentioned in the reference genome is used to analyse the spike glycoprotein region particularly. The masked entropy of all the sites in spike glycoprotein region is separately plotted. The spike glycoprotein contains an RGD motif. Several integrins have a high affinity for RGD motifs. So, the site of the RGD motif present in the spike protein is traced using reference genome and the information entropies of the sites having RGD motif are observed. The masked entropy values for the sites coding for the RGD motif are shown in **Supplementary Table 1**.

*Selection of ISM sites*

To select the sites for ISMs, we have used NULL frequency as a parameter. NULL frequencies of all the sites are calculated as number of ‘N’ or ‘-’ at a position in all the aligned sequences divided by the total number of these sequences. We plot the count of positions having particular NULL frequency values against their NULL frequencies in **Supplementary Figure 2b**. We use the calculated masked entropy to plot the count of positions having particular masked entropy values against their masked entropies. We use these plots to set the thresholds for the parameters to select sites for ISMs. The sites with high number of ‘N’ or ‘-’ do not have high information (or high mutation) and cannot be used to create ISM, so we observe the distribution of masked entropy and NULL frequency across the sites in **Supplementary Figure 2a** and **2b** respectively.

*Progression of mutations*

The 50 most abundant unique ISMs which are used for heirarchical clustering in **Figure 2** are also used to observe the overall progression of the mutations in SARS-CoV-2. In **Supplementary Figure 3**, the hamming distances of these 50 ISMs from the reference ISM are calculated and are plotted against the number of days from the reference date to the date of their first incidence. To compare this progression in mutation with the rise in worldwide cases, the total cases are also plotted against the number of days since the reference date on the same plot.

*Relative abundance of ISM*

The relative abundance of an ISM in a region standardly corresponds to the fraction of that ISM sequenced among all the ISMs sequenced in that region. The relative abundance of India’s most abundant ISM *TCTGTCGGAAC* is shown in **Supplementary Figure 4**. The geographical distribution of ISMs across different states and UTs in India is shown in **Supplementary Figure 6,**

#### *Phylodynamic and Phylogeographic Analyses*

The phylodynamic and phylogeographic analyses was performed on the 798 unique ISMs using BEAST2 tool. The dates of first appearance were used as tip dates for these ISMs. The latitudes and longitudes of the countries were used as location points for phylogeographic analysis.

The phylodynamic analysis was done using default settings (95% highest posterior density interval [HPD 95%]) for HKY Gamma Site Model, Strict Clock Model and Coalescent Exponential Population Priors and default parameters for MCMC. After running BEAST2 tool, tree file was processed to make maximum clade credibility (MCC) tree using BEAST2’s TreeAnnotator app on default parameters. **Supplementary Figure 7** shows the MCC tree plotted using FigTree v1.4.4 with the calculated time of the respective nodes.

The phylogeographic analysis was done using default settings for HKY Gamma Site Model (with empirical frequencies) and Coalescent Constant Population Priors. The Strict Clock Model was used with a clock rate of 2.0 × 10^-5^ ­to speed up convergence. A separate special geography partition was added with default settings for HKY Gamma Site Model, Relaxed Clock Log Normal Clock Model and Coalescent Constant Population Priors. MCMC with 10^6^ chain length (default chain length = 10^7^) was used. After running BEAST2 tool, tree file was processed by TreeAnnotator to make maximum clade credibility (MCC) tree. To improve visualization and prevent overcrowding of the output figure for phylogeography, TreeAnnotator was used on Burnin Percentage =10 and Posterior Probability Limit=0.7 with other parameters as default. This was followed by generation of the final phylogeographic plot using SPREAD v1.0.6 which is shown in **Supplementary Figure 8**.

*Relative abundance of strains*

The relative abundance of a strain in a region is evaluated as the fraction of that strain sequenced among all the samples sequenced in that region. The geographical distribution of strains across different states and UTs in India shown in **Supplementary Figure 9** uses relative abundance for visualization.

The temporal analysis shows the progression of the SARS-CoV-2 subtypes as a function of time. The dates associated with the sequences is used to find out the relative abundance of the viral subtypes at a point of time in a country through the course of time since a given point of time. The relative abundance *ISM_(s,c)_(t)* of a subtype *s*, in a region *c*at a given point of time *t,* is calculated as:

$${ISM}_{(s, c)}(t) = \frac{N_{(s,c)}(t)}{N_{c}(t)}$$

Where, *N_(s,c)_(t)* indicates total number of instances of subtype *s* in a region *c* till a point of time *t*, and *N_c_(t)*is the total number of sequences in the location *c* till a point of time *t.* The temporal analysis of relative abundance of strains sequenced in India is shown in **Supplementary Figure 10**.

**Supplementary Tables**

**Supplementary Table 1.** Show the masked entropy values of region coding for RGD motif.

| **Bases** | **Position** | **Masked Entropy** |
| --- | --- | --- |
| A | 39680 | 3.96×10^-4^ |
| G | 39681 | 2.76 ×10^-3^ |
| A | 39682 | 1.98 ×10^-4^ |
| G | 39683 | 5.95 ×10^-4^ |
| G | 39684 | 9.38 ×10^-4^ |
| T | 39685 | 1.98 ×10^-4^ |
| G | 39686 | 1.98 ×10^-4^ |
| A | 39687 | 1.98 ×10^-4^ |
| T | 39688 | 1.98 ×10^-4^ |

**Bases**: nucleotide bases coding for RGD motif; **Position**: base-pair position in the reference genome post multiple sequence alignment; **Masked Entropy**: masked entropy value at respective ISM position.

**Supplementary Figures**


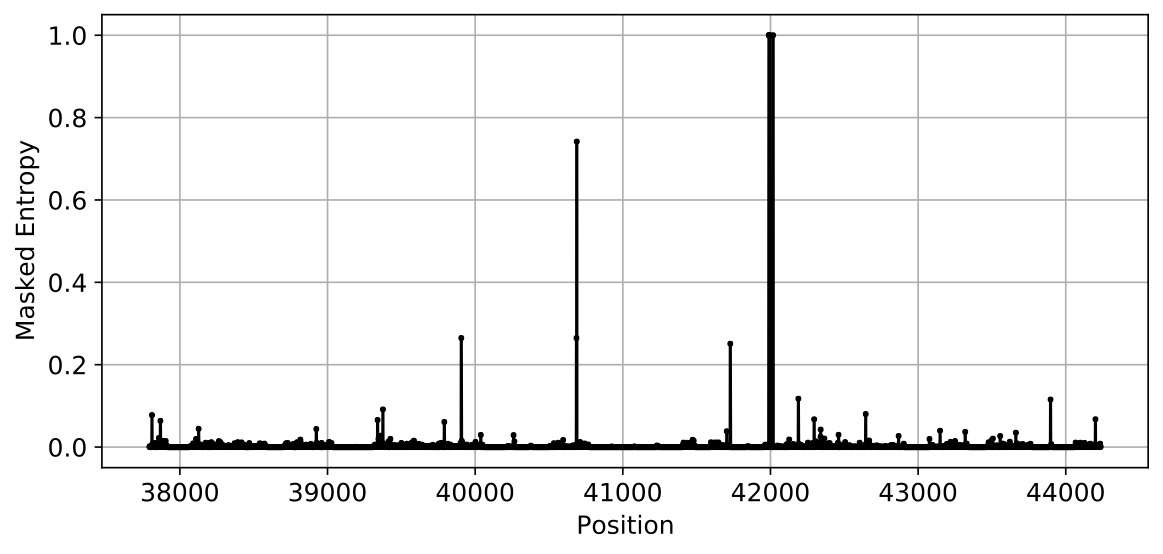


**Supplementary Figure 1:** Shows masked entropies of different positions in the spike glycoprotein region of the SARS-CoV-2 genome. There are a few positions with high masked entropy (entropy> 0.4). The position 40689 with masked entropy 0.74 is used for constructing ISMs (see Table 1 in main text). The other positions near 42000 also shows a very high entropy but they are not selected for ISM owing to their high null frequency.


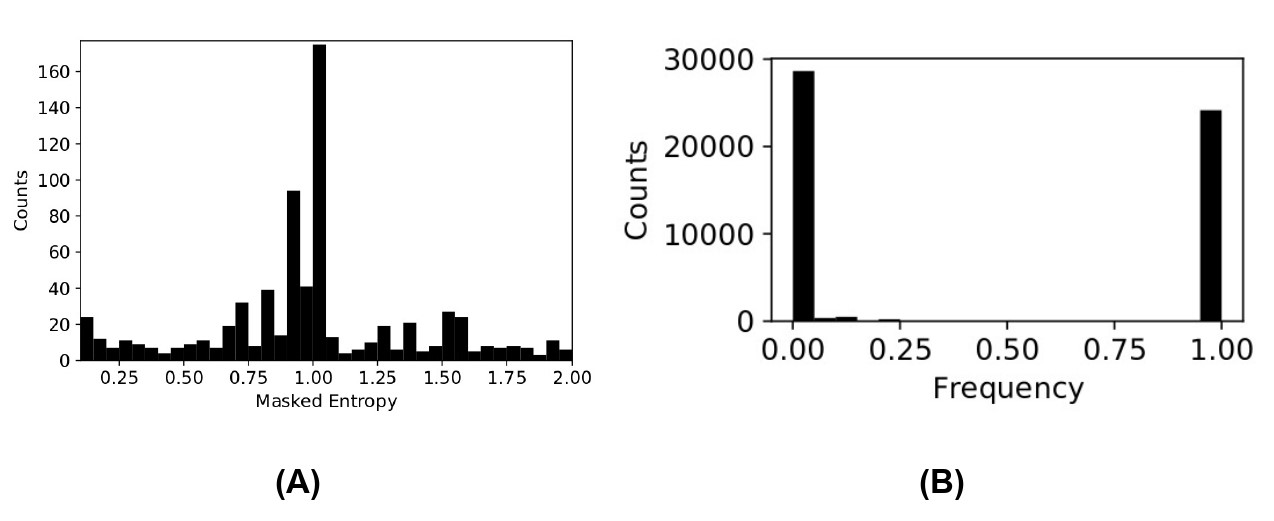
**Supplementary Figure 2:** The histogram of a) masked entropies and, b) null frequencies at different positions of SARS CoV-2 genome. These distributions were used to set thresholds for masked entropy and null frequency values for selection of positions for ISMs.


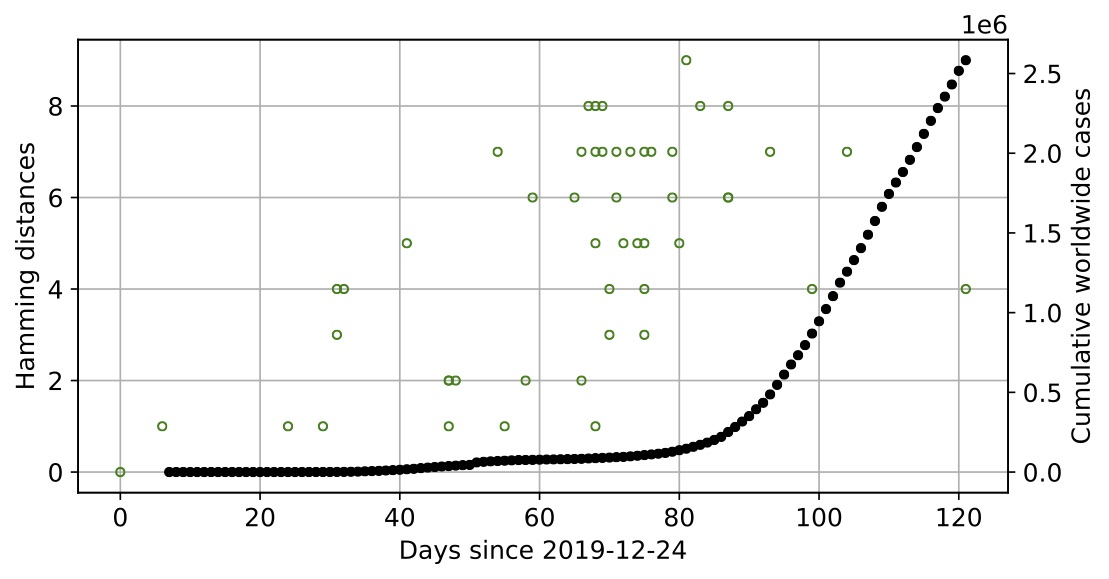


**Supplementary Figure 3:** Shows overall progression of mutations in SARS-CoV-2 genome as a function of the number of COVID-19 cases worldwide. The green circle refers to an ISM along with their first sequenced date (on horizontal axis) and its hamming distances (left vertical axis). The black filled circles indicates the number of COVID-positive cases (in millions, right vertical axis). The hamming distances were computed using 50 most abundant ISMs with the reference ISM.


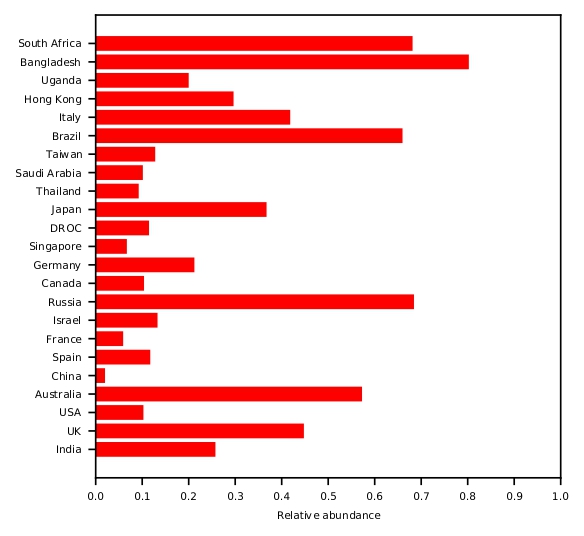


**Supplementary Figure 4:** Shows the relative abundance of ISM *TCTGTCGGAAC* in different countries. This ISM was identified to be the top most abundant in India (25.7%). The red colour chosen for distribution of this ISM is as per the global color map (see Supplementary Figure 5).


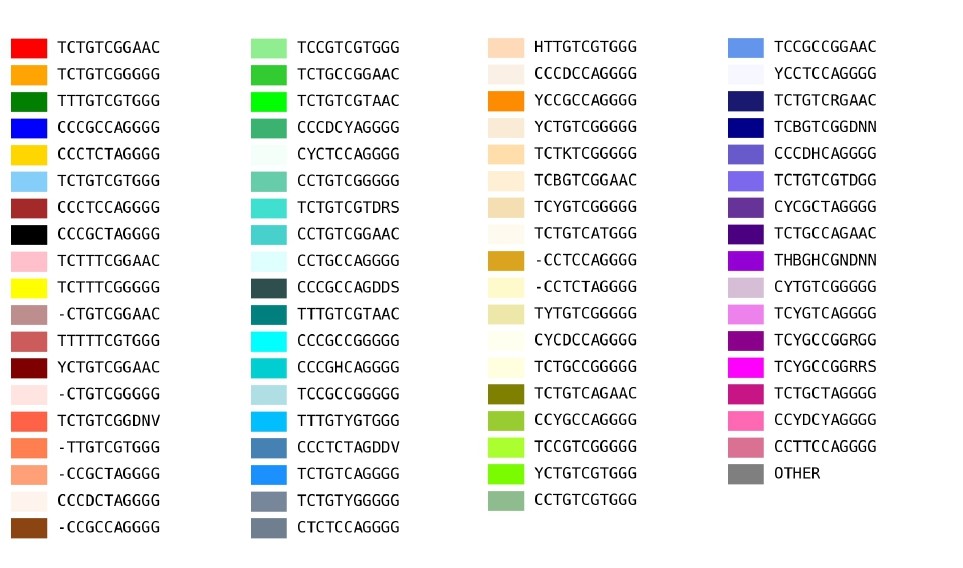


**Supplementary Figure 5:** Shows the global color map used throughout in this study for different viral subtypes as represented by unique ISMs.





**Supplementary Figure 6:** Shows the geographical distribution of different viral subtypes across different states or union territories (UTs) in India. The legend box beside the pie chart indicates the ISM’s first sampling date in that region. The color key at the bottom shows the colour map for ISMs corresponding to viral subtypes. The viral subtypes highly abundant in India corresponding to red (*TCTGTCGGAAC*; 25.7%), yellow (*TCTGTCGGGGG*; 20.7%), blue (*TCTGTCGTGGG*; 18.3%) and brown (*CCCTCCAGGGG*; 14.7%) contribute to most of the states and UTs.

**SEE PDF for Supplementary Figure 7**

**Supplementary Figure 7:** Shows the maximum clade credibility (MCC) tree for the phylodynamic analysis of the 798 unique ISMs. The taxa shown in the figure provide details of the sequence, date and country of first appearance for each ISM. The estimated TMRCA (equal to 2019.4383; June 9^th^, 2020) is highlighted at the root node.


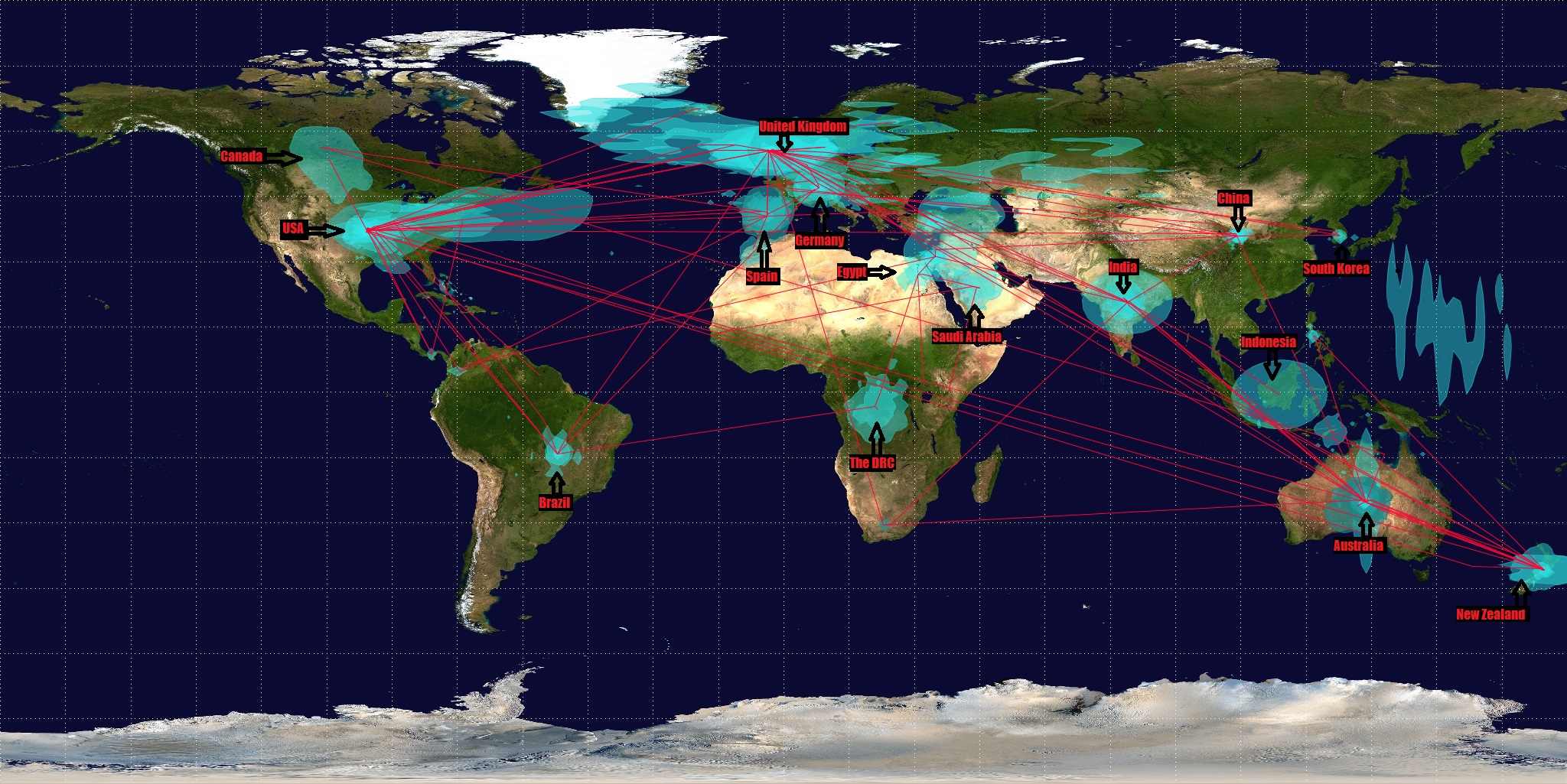


**Supplementary Figure 8:** Shows the phylogeography of 798 unique identified ISMs. It shows the branches of the MCC tree for phylogeographic analysis which have their color from black to red as per their node height. The circular polygons shown in cyan color show a discrete state in the MCC tree where the radius and color (black to cyan) indicate the number of lineages holding that discrete state at a time. The major countries highlighted by the circular polygons have been marked with their name.





**Supplementary Figure 9:** Shows geographical distribution of different strains across different states or UTs in India. The legend at right bottom mentions the colours corresponding to different strains. ‘D’ and ‘G’ refer to the S-D614 and S-G614 strains respectively. ‘OTHER’ refers collectively to the strains with abundance less than 0.5% and ‘-’ refers to the samples which could not code for an amino acid at position 614 of the S-protein.


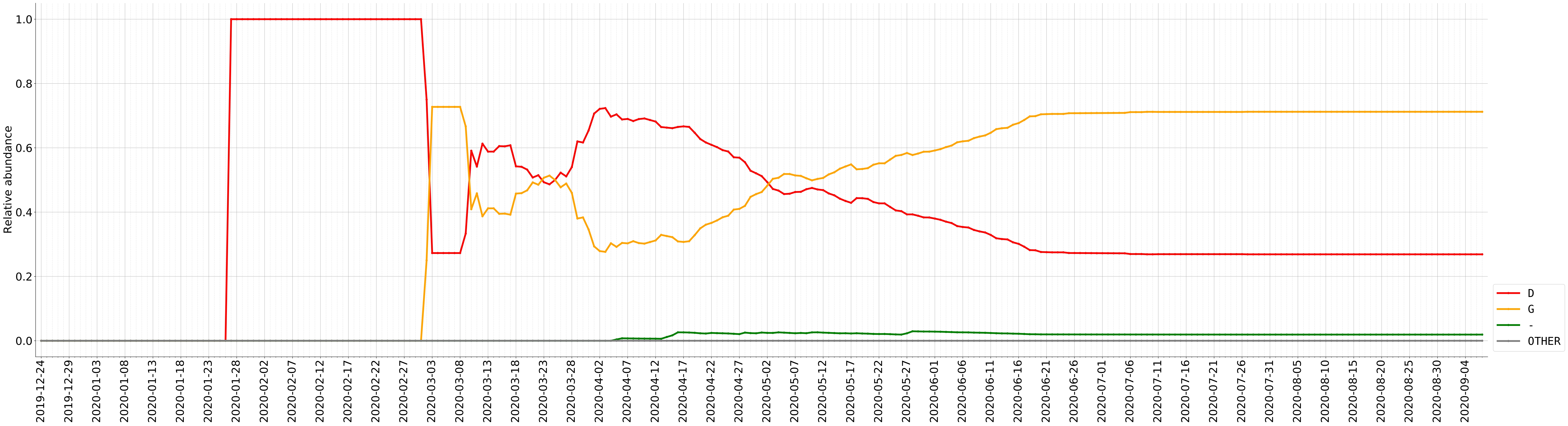


**Supplementary Figure 10:** Show the relative abundance of strains of genome sequences from India as sampled over time.
