# Supplementary material for: Entropy based analysis of SARS-CoV-2 spread in India using informative subtype markers
Source: Sci Rep. 2021 Aug 5;11:15972. doi: 10.1038/s41598-021-95247-5 (PMC8342543; doi:10.1038/s41598-021-95247-5)
Supplement: Supplementary file 2 — Supplementary Information 2. [file 41598_2021_95247_MOESM2_ESM.pdf]

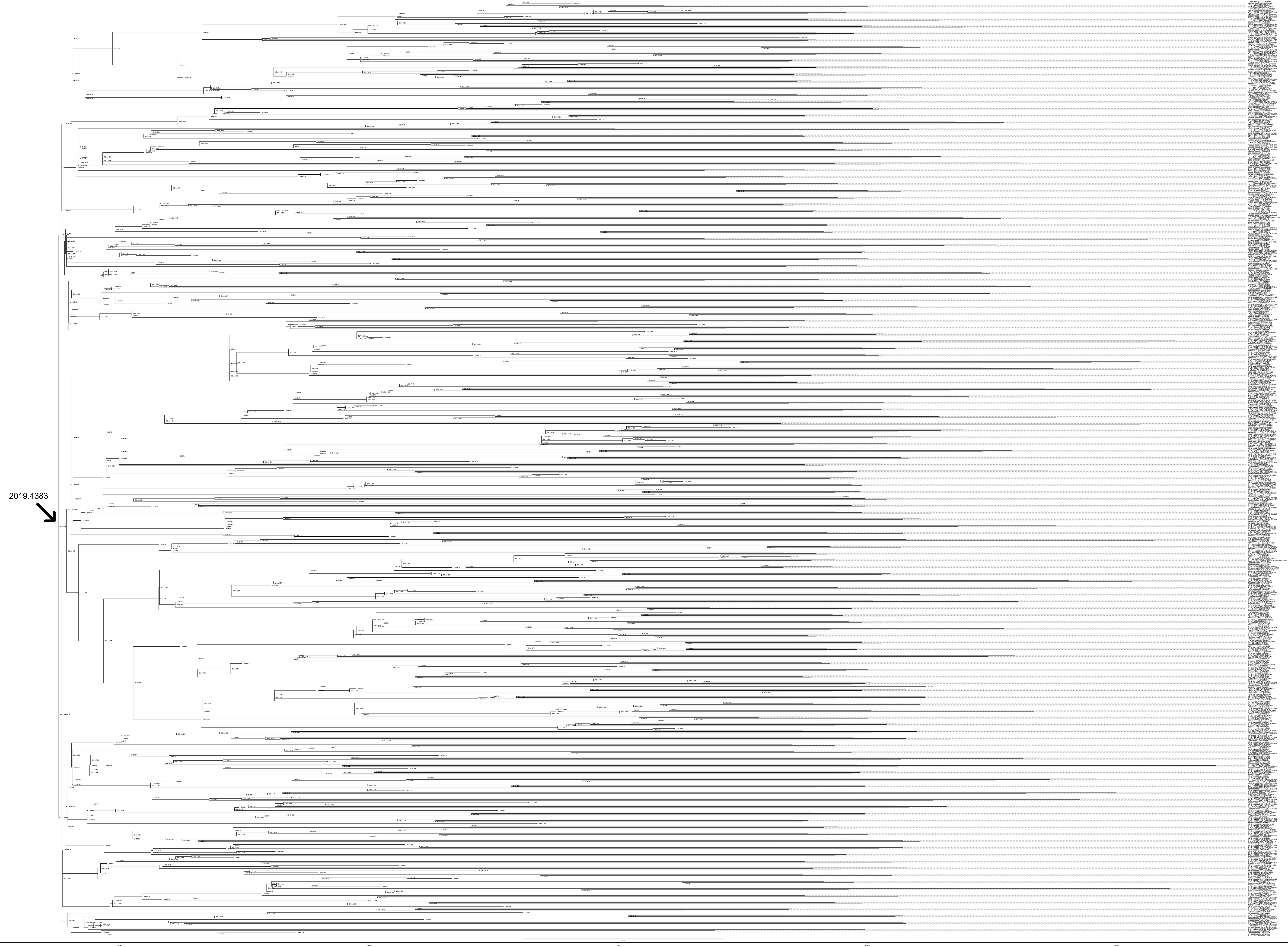
2019.4383
